# Proteomic analysis of *Pteropus alecto* kidney cells in response to the viral mimic, Poly I:C

**DOI:** 10.1186/s12953-015-0081-6

**Published:** 2015-11-02

**Authors:** Lawrence Mok, James W. Wynne, Kris Ford, Brian Shiell, Antony Bacic, Wojtek P. Michalski

**Affiliations:** CSIRO, Australian Animal Health Laboratory, East Geelong, 3219 VIC Australia; ARC Centre of Excellence in Plant Cell Walls, School of BioSciences, The University of Melbourne, Parkville, VIC Australia; Bio21 Institute for Molecular Science and Biotechnology, The University of Melbourne, Parkville, VIC Australia

**Keywords:** Quantitative proteomics, DIGE, iTRAQ, Bat cells, Poly I:C, Viral mimic, dsRNA analogue

## Abstract

**Background:**

Bats are recognised as an important reservoir for a number of highly pathogenic zoonotic viruses. While many of these viruses cause severe and often fatal disease in humans, bats are able to coexist with these viruses without clinical signs of disease. The mechanism conferring this antiviral response is not fully understood. Here, we investigated the differential protein expression of immortalised *Pteropus alecto* kidney cells (PaKiT03) following transfection with the viral mimic, Poly I:C. Two complementary proteomic approaches, difference gel electrophoresis (DIGE) and isobaric tagging for relative and absolute quantitation (iTRAQ) were used to quantify changes in protein expression following Poly I:C stimulation at 4, 8 and 20 hr post treatment (hpt).

**Results:**

The expression of *ISG54* gene, a known responder to virus infection and Poly I:C treatment, was significantly induced in transfected cells compared with mock-transfected cells. Through iTRAQ analysis we show that Poly I:C up-regulates key glycolytic enzymes at 4 hpt within PaKiT03 cells. In contrast, at 20 hpt PaKiT03 cells down-regulated ribosomal subunit proteins. The analysis with DIGE of Poly I:C transfected PaKiT03 cells showed over 215 individual spots differentially regulated, however only 25 spots could be unambiguously identified by LC-MS/MS. Immunoblotting confirmed the up-regulation of Eno1 and Tpi1 in PaKiT03 cells following Poly I:C transfection. A comparison with human cells (HEK293T and HeLa) and one additional bat cell line (PaLuT02), demonstrated that glycolytic pathways are also induced in these cell types, but at different intensities.

**Conclusion:**

The two techniques, DIGE and iTRAQ identified largely overlapping sets of differentially expressed proteins, however DIGE unambiguously identified significantly less proteins than iTRAQ. Poly I:C induced a rapid metabolic shift towards glycolysis within the PaKiT03 cells at 4 hpt, presumably as a consequence of increased energy requirements. On the other hand ribosomal subunit proteins were seen as down-regulated by iTRAQ, these proteins may be the limiting factors in the translational machinery available for virus replication. This study provides new insight into the antiviral response of bat cells, highlighting the importance of energy metabolism.

**Electronic supplementary material:**

The online version of this article (doi:10.1186/s12953-015-0081-6) contains supplementary material, which is available to authorized users.

## Background

Bats are the natural reservoir for a number of emerging and re-emerging viruses including severe acute respiratory syndrome (SARS)-like and Middle East respiratory syndrome (MERS) coronaviruses (CoV) [1, 2], Hendra and Nipah paramyxoviruses [3, 4], and the filoviruses, Ebola and Marburg [5, 6]. The spill-over of these viruses from bats to humans, often through an intermediate host, can cause severe and fatal disease in humans. Recent high profile examples include the global SARS epidemic in 2003, which caused the deaths of over 800 people. Investigations led by two independent groups both demonstrated that the natural reservoir for the SARS-like CoV were bats of the genus *Rhinolophus* [1, 7]. More recent examples of spill over events from bats to humans include the 2014 Ebola virus epidemic in West Africa that is believed to be of bat origin [8, 9].

While many bat borne pathogens cause severe and often fatal diseases in humans, bats demonstrate no clinical signs of disease when infected with these agents. Indeed, experimental infections of bats with highly pathogenic viruses such as Hendra and Nipah virus yielded no observable clinical signs. However, virus isolation, seroconversion, and the excretion of virus in saliva, urine and faeces were observed [10, 11]. Subclinical infections of both fruit and insectivorous bats have also been reported following experimental infection with Zaire Ebola virus. High titres of Ebola virus were successfully obtained from viscera and faecal samples following experimental infection [12].

A multitude of protective responses are invoked following the infection of a cell from both the innate and adaptive immune systems. One of the early innate responses is the induction of interferons (IFNs) which exert their effects through the transcription of a large set of interferon stimulated genes (ISGs) [13]. The products of these genes have many functions ranging from directly acting on the virus via interfering with virus uncoating to modulating key functions within the host cells such as inhibiting protein translation and apoptosis [14]. Beside these known innate processes, there may be others that still await identification and elucidation. Previous studies on bats have focused on genome sequencing, transcriptomics and the investigation of specific components of the innate and adaptive immune system, such as pattern recognition receptors, antibody diversity and IFNs [15–18]. Important resources generated from these studies include the genome sequences of nine bats species [15, 19–21] and immortalised cell lines for *in vitro* studies [22]. The investigation of bat immunoglobulins identified IgG and IgM in bat serum but IgA was only detected in trace quantities and the higher quantities of IgG in mucosal secretions is thought to compensate for the lower abundance of IgA [23]. All these studies have shown that bats possess genes present in other mammalian species, including components of the innate and adaptive immune system [16]. Functional studies of bat IFNs show an induction of IFN genes and the subsequent antiviral activity following virus infection [24]. In terms of proteomics research, little has been studied in this area. We have previously identified that Hendra virus infection of *P. alecto* kidney cells sensitises these cells to TRAIL-mediated apoptosis [25]. Despite these efforts the exact mechanisms by which bats manage virus infection is yet to be identified.

There are a number of different proteomic methodologies that are used for quantitative analyses or proteome expression. Fundamentally, these can be grouped as either gel-based or gel-free methods. In gel-based techniques protein separation is achieved by electrophoresis (1-D or 2-D) and separated proteins are stained or labelled and the intensities of protein bands (1-D) or spots (2-D) are quantified prior to protein identification by mass spectrometry (MS). In gel-free techniques the quantitative data and protein identities are obtained from the mass spectra of differentially labelled proteins. Both of these approaches have been used to study the host proteome in response to virus infection [26–28].

Here, we undertake a comparative proteomic study utilising two different quantitative proteomic techniques in parallel, namely *di*fference *g*el *e*lectrophoresis (DIGE) [29] and *i*sobaric *t*agging for *r*elative and *a*bsolute *q*uantitation (iTRAQ) [30] to analyse the proteome of immortalised *P. alecto* kidney cells (PaKiT03) [22] challenged with polyinosinic:polycytidylic acid (Poly I:C). Poly I:C is an analog to dsRNA and has been used to stimulate cells and induce a potent antiviral cellular responses [31]. It is recognised by a number of sensory molecules of the cell, termed pathogen recognition receptors [32] resulting in the production of numerous cytokines that in turn induce a number of immune pathways [31]. Although the main purpose of this study was to generate a proteomic dataset as a useful resource for future research in bat immunology, we were also interested in assessing the advantages and limitations of two commonly used proteomic methodologies.

## Results

### PaKiT03 cells are responsive to Poly I:C transfection

**Fig. 1:**
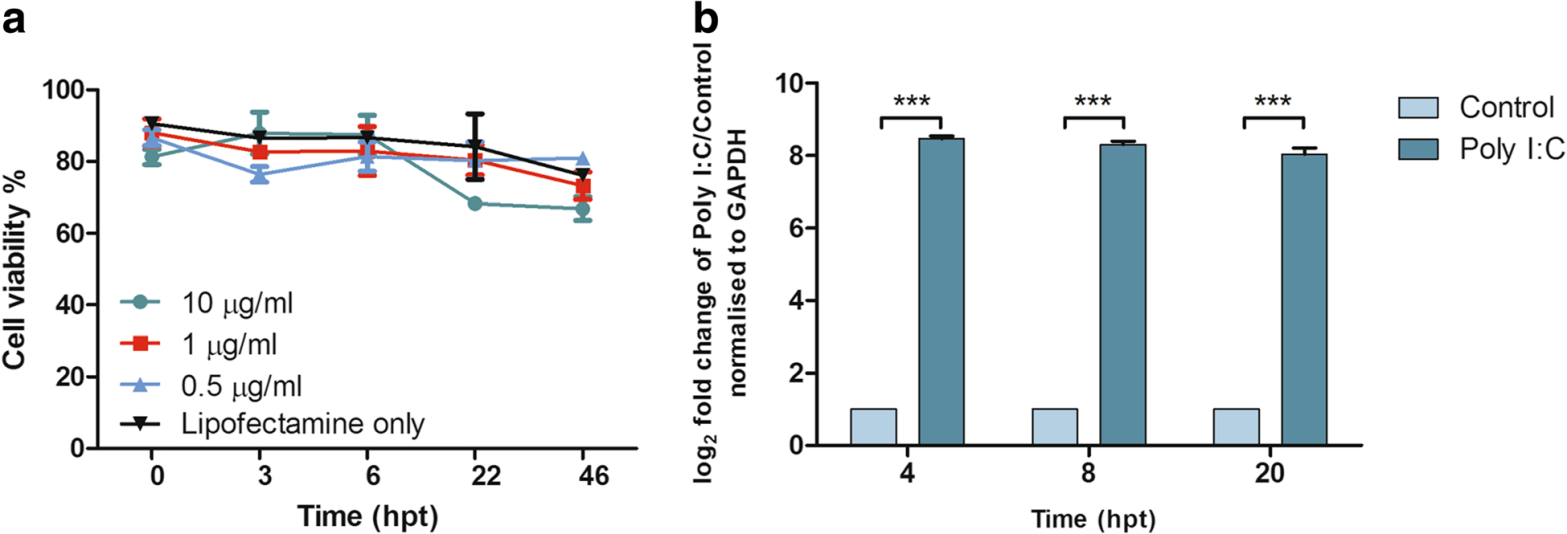
Response of cells to Poly I:C. **a** Viability of PaKiT03 cells following transfection with 0.5, 1 and 10 μg/ml of Poly I:C with Lipofectamine 2000 and Lipofectamine 2000 only. **b** Relative expression of *ISG54* in PaKiT03 cells transfected with 1 μg/ml of Poly I:C over transfected control and normalised to the housekeeping gene *GAPDH*

The viability of PaKiT03 cells was assessed at 3, 6, 22 and 46 hr following transfection with increasing concentrations of Poly I:C (0.5, 1 or 10 μg/ml) delivered with Lipofectamine. An initial decrease in viability was observed in all cells following transfection (Fig. 1a). From 6 to 22 hr post transfection (hpt) the viability of cells treated with 0.5 μg/ml, 1 μg/ml of Poly I:C or with Lipofectamine alone remained stable while the viability of cells treated with 10 μg/ml of Poly I:C dropped to below 70 %. At 46 hpt the viability for all cells was found to be at 75–80 % except for those treated with 10 μg/ml of Poly I:C, which were still below 70 % viability. Based on these findings it was decided that transfection with 1 μg/ml of Poly I:C was the least detrimental to cell viability and thus most appropriate for further studies.

To ensure that cells were responsive to Poly I:C, the induction of interferon stimulated gene 54 (*ISG54*) was measured using quantitative real-time PCR. Up-regulation of *ISG54* is a known response to virus infection and has been linked to apoptosis [33]. The relative expression of *ISG54* mRNA in PaKiT03 cells following Poly I:C transfection was significantly up-regulated in cells transfected with 1 μg/ml of Poly I:C at 4, 8 and 20 hpt compared to the non-transfected control (Fig. 1b).

### iTRAQ

**Fig. 2:**
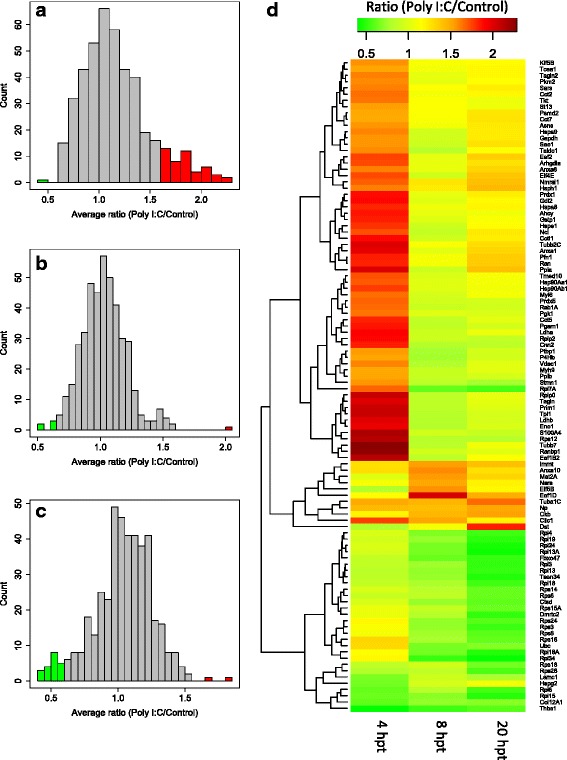
Analysis of iTRAQ data. **a-c** The average ratio of Poly I:C/Control of three biological replicates at 4 hpt (a), 8 hpt (b) and 20 hpt (c). Proteins with fold-change ≥ 1.5 are shown in red (up-regulated) and green (down-regulated). **d** Heatmap of 104 differentially regulated proteins at 4, 8 and 20 hpt

For iTRAQ analysis, mass spectra from samples obtained at the three time points were searched against the translated *P. alecto* genome using MASCOT and ProteinPilot. Three datasets were generated from the three biological replicates. In total, 426 proteins were identified across the three time points with ≥ 2 peptide matches. Of the 426 proteins identified 104 were differentially expressed, based on mean fold-change of ≥ 1.5. The number of differentially expressed proteins varied between time points (Table 1). At 4 hpt the majority of differentially expressed proteins were up-regulated (Fig. 2a). At 8 hpt, only a small number of proteins were differentially expressed, with an almost equal number of proteins up and down-regulated (Fig. 2b). In contrast, at 20 hpt most differentially expressed proteins were down-regulated (Fig. 2c). Illustrated by the heatmap (Fig. 2d), proteins that were up-regulated by Poly I:C at 4 hpt, did not remain up-regulated at 8 and 20 hpt. However, many of the proteins down-regulated by Poly I:C transfection at 20 hpt were also down-regulated at 8 hpt. A full list of protein expression profiles obtained from iTRAQ analysis is provided as Additional file 1.

**Table 1:** Summary statistics of proteins/spots differentially expressed in PaKiT03 cells following Poly I:C stimulation at 4, 8 and 20 hpt

Method	Regulation	4 hpt	8 hpt	20 hpt
iTRAQ	Up	66	7	4
	Down	6	8	26
DIGE (spots)	Up	0	6	125
	Down	0	7	90

### DIGE

**Fig. 3:**
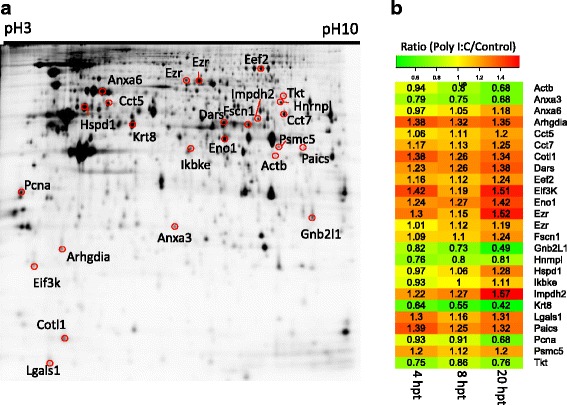
Separation of PaKiT03 proteins and DIGE. **a** Extracted PaKiT03 proteins separated by 2DE using a 3–10 nonlinear immobilized pH gradient strip and 12.5 % poly-acrylamide gel. Numbered gel spots highlighted in red circles were manually excised and unambiguously identified by LC-MS/MS. **b** Heatmap showing the expression ratio (Poly I:C/Control) of the 25 differentially expressed protein spots with unambiguous identities at 4, 8 and 20 hpt

Triplicate cell lysates from PaKiT03 cells transfected with Poly I:C for 4, 8 and 20 hr were also analysed by DIGE (Fig. 3a). A total of 2,385 protein spots were present in all gels. Of the 2,385 spots, 215 (9 %) were differentially expressed (*p* ≤ 0.05). The majority of differential expression was observed at 20 hpt with 125 spots up-regulated and 90 spots down-regulated (Table 1). Spots of interest were identified using liquid chromatography-mass spectrometry (LC-MS). A total of 42 spots that were differentially expressed were manually excised from gels and processed for LC-MS. Of these protein spots, 25 returned a single protein identity, while 17 yielded two or more identities. The 17 spots with ambiguous protein identities were removed from further analysis. The regulation of the 25 unambiguously identified proteins was assessed across the three time points (Fig. 3b) with the majority of these proteins up-regulated. In general, proteins followed the same regulation at each time point. Proteins that were up-regulated at 4 hpt were also up-regulated at 8 and 20 hpt. This pattern of regulation was also observed for proteins that were down-regulated. A comparison of the iTRAQ and DIGE datasets revealed significant overlap. Of the 25 proteins unambiguously identified by DIGE, 18 (72 %) were also detected within the iTRAQ analysis.

### PolyI:C up-regulates proteins within the glycolytic metabolism pathway

Gene ontology (GO) enrichment analysis was performed on lists of up and down-regulated proteins. Here, we focused on those proteins found to be differentially expressed by iTRAQ, as too few proteins were identified by DIGE to perform GO enrichment analysis. Significant enrichment for proteins involved in the glycolytic metabolism pathway was observed in the up-regulated protein list (Table 2). Indeed, GO terms such as pyruvate metabolic process (GO:0006090) and glycolytic process (GO:0006096) were significantly enriched within the up-regulated protein list. Individual proteins that contributed to this pathway included Ldhb, Pgk1, Gapdh, Tpi1, Ldha, Pgam1, Pkm and Eno1. In contrast, a strong enrichment for ribosomal processes, including translational termination (GO:0006415) was observed in the down-regulated protein list (Table 2). Contributing to this enrichment was the down-regulation of over 20 different ribosomal proteins at 20 hpt.

**Table 2:** Gene Ontology enrichment of up- and down-regulated protein lists. The most significant five enriched GO classes are shown for the up- and down-regulated proteins

Regulation	GO ID	Gene Ontology	FDR q-value	Proteins contributing
Up-regulated	GO:0006090	pyruvate metabolic process	6.40E-01	Ldhb, Pgk1, Gapdh, Tpi1, Ldha, Pgam1, Pkm, Eno1
	GO:0006733	oxidoreduction coenzyme metabolic process	3.49E-01	Ldhb, Pgk1, Gapdh, Tpi1, Ldha, Pgam1, Pkm, Eno1, Taldo1, Tkt, Prdx5
	GO:0046496	nicotinamide nucleotide metabolic process	1.40E-01	Ldhb, Pgk1, Gapdh, Tpi1, Ldha, Pgam1, Pkm, Eno1, Taldo1, Tkt, Prdx5
	GO:0006757	ATP generation from ADP	4.47E-01	Pgk1, Gapdh, Tpi1, Ldha, Pgam1, Pkm, Eno1
	GO:0006096	glycolytic process	3.84E-01	Pgk1, Gapdh, Tpi1, Ldha, Pgam1, Pkm, Eno1
Down-regulated	GO:0006415	translational termination	1.31E-18	Rps24, Rpl24, Rps3, Rpl19, Rpl18A, Rpl18, Rps16, Rpl13A, Rps15A, Rpl15, Rpl13, Rps18, Rpl7A, Rps8, Rpl34,Rps14, Rpl6, Rps28, Rpl4, Rps6, Rpl3
	GO:0022411	cellular component disassembly	2.45E-18	Same as above
	GO:0043624	cellular protein complex disassembly	3.79E-18	Same as above
	GO:0006614	SRP-dependent cotranslational protein targeting to membrane	7.66E-18	Same as above
	GO:0006613	cotranslational protein targeting to membrane	6.13E-18	Same as above

### Immunodetection of differentially regulated proteins

**Fig. 4:**
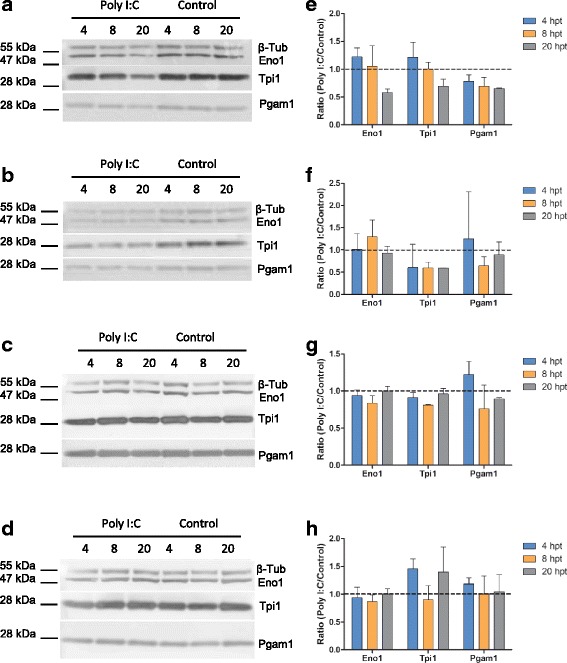
Immunodetection of glycolytic enzymes within bat and human cells. **a-d** Immunodetection of Eno1, Tpi1, Pgam1 and β2-Tub (load control) in Poly I:C transfected and Control cells at 4, 8 and 20 hpt (a: PaKiT03, b: PaLuT02, c: HEK293T, d: HeLa). **e-h** Densitometry analysis following immunodetection of Eno1, Tpi1, Pgam1. Average ratios were calculated as Poly I:C/Control normalised to β-Tub. Error bars represent the standard error of the mean from two technical replicates (e: PaKiT03, f: PaLuT02, g: HEK293T, h: HeLa)

We further assessed the response of three glycolytic enzymes, α-enolase (Eno1), phosphoglycerate mutase 1 (Pgam1) and triosephosphate isomerase 1 (Tpi1) to Poly I:C across two human (HEK293T and HeLa) and two bat cell lines (PaKiT03, PaLuT02) using immunodetection. Polyclonal antibodies specific to Eno1, Pgam1 and Tpi1, successfully detected their protein of interest in both bat (Fig. 4a, b) and human cell lines (Fig. 4c, d). Using β-Tubulin as an internal load control, we calculated the ratio of Eno1, Pgam1 and Tpi1, as Poly I:C:Control normalised to β-Tubulin (Fig. 4e-f). In PaKiT03 cells it was shown that both Eno1 and Tpi1 are induced in Poly I:C transfected cells at 4 hpt, and then decreased at 20 hpt. Eno1 was also induced in the PaLuT02 cells at 8 hpt, but Tpi1 was down-regulated at all time points in PaLuT02 cells. The PaLuT02 cells also show a small up-regulation of Pgam1 at 4 hpi. The magnitude of differential expression was lower than that observed in the iTRAQ analysis for all proteins. Poly I:C induced Tpi1 in HeLa, but not HEK293T cells. Interestingly, this up-regulation was observed only at 4 and 20 hpt, but not 8 hpt. Both human cell lines show a small increase in Pgam1 expression at 4 hpt, similarly observed in the PaLuT03 cells at 4 hpt. Poly I:C did not up-regulate Eno1 in either HeLa or HEK293T cells at any time point.

## Discussion

Bats co-exist with a diverse range of highly pathogenic viruses. High profile examples include the henipaviruses, filoviruses and SARS/MERS coronaviruses. While these viruses cause significant disease in humans, bats do not show signs of clinical disease following infection. We hypothesise that the unique host-pathogen interplay between bats and viruses may hold significant insight into novel and/or atypically anti-viral mechanisms. With this in mind, the present study examined the response of cultured bat kidney cells to the viral mimic Poly I:C. The use of Poly I:C, has a number of advantages over live virus infection. Firstly, it reduces the heterogeneity associated with viral infection in cell culture. During live virus infection there may only be a small proportion of cells that become infected and at a given time point, thus uninfected cells may obscure or dilute the observable response. Secondly, many viruses antagonise the immune response and thus mask potentially unknown and more subtle antiviral responses [34–36]. Therefore Poly I:C provides a simple and controlled mimic for RNA virus infection and has been used previously to evaluate antiviral responses [37, 38].

Analysis of iTRAQ proteins up-regulated at 4 hpt demonstrated an enrichment of proteins related to the glycolytic metabolic pathway. Proteins such as Eno1, Pgk1, Gapdh, Tpi1, Ldha, Pgam1, Pkm and Ldhb, show an increased expression in the PaKiT03 at 4 hpt, which suggests an increase in energy requirements. Understandably, the initiation of an immune response is energetically taxing and requires the cell to synthesise a wide range of molecules such as ISG products and cytokines that serve to defend against the invading pathogen. The induction of Eno1, Pgk1, Gapdh, Tpi1, Pgam1 and Pkm at 4 hpt suggests a rapid shift towards glycolysis early in the anti-viral response in PaKiT03 cells. Glycolysis, the conversion of glucose into pyruvate, consists of multiple steps that require key glycolytic enzymes. Tpi1, Gapdh, Pgk1, Pgam1, Eno1 and Pkm, are the key enzymes that are essential for steps 5, 6, 7, 8, 9 and 10 in the glycolysis pathway [39]. Other glycolytic enzymes that were identified, but not differentially expressed, in our iTRAQ data included Hk1, Pfkp and Aldoa. These enzymes control steps 1, 3 and 4 of the glycolysis pathway [39].

Immunodetection confirmed the up-regulation of Eno1 and Tpi1 at 4 hpt within the PaKiT03 cells, albeit it to a lesser magnitude than quantified by iTRAQ analysis. In contrast, immunodetection demonstrated a down-regulation of Pgam1 in PaKit03 cells at 4 hpt. In comparison, both human cell lines and the bat lung cell line (PaLuT02) showed up-regulation of Pgam1 at 4 hpt. HeLa cells also showed up-regulation of Tpi1 at 4 and 20 hpt. These findings suggest that the induction of glycolytic pathways are not specific to PaKiT03 cells, however the kinetics of key glycolytic enzymes varies between cell lines. Previous study in mice have shown that Poly I:C induced type I IFN, rapidly promoting a metabolic shift from oxidative phosphorylation to glycolysis and is required for the maturation of dendritic cells [40] Viruses have also been shown to induce metabolic pathways. Indeed, Hepatitis C virus protein expression has been shown to preferentially promote nonoxidative glucose metabolism over oxidative phosphorylation pathways in human cell lines [41]. Similarly Herpes simplex virus type 1 was shown to induce key glycolytic enzymes and induced glycolysis in Vero cells [42].

In addition to the metabolic proteins that were up-regulated, we also identified a large number of ribosomal subunit proteins which were found to be down-regulated at 20 hpt. The decrease in levels of these ribosomal proteins by the host cell is a possible defence to virus infection. As not all of the components required for virus replication is encoded by the viral genome, viruses are known to hijack and utilise the host ribosome for the translation of viral proteins. This mechanism has been shown to be utilised by HIV, by binding the 40S ribosomal protein [43, 44]. Limiting access to this machinery may be used as a means to slow down replication of virus in infected cells.

Poly I:C is known to induce ISGs, however we were unable to detect any protein expression of ISGs within our study. It is likely that the absence of these ISGs in our datasets is related to the relative abundance of these proteins in the cell. The dynamic range for cellular proteins spans at least seven orders of magnitude [45] and iTRAQ suffers from low detection sensitivity with high abundance proteins masking low abundance proteins. The enrichment of these proteins by fractionation of the sample may result in the improved detection and the identification of these immune effector proteins. Due to technical limitations involved in spot picking we found DIGE to be unsuitable for whole cell lysate proteomics. In many cases, DIGE protein spots are simply too close to one another to successfully isolate a single spot. In comparison, iTRAQ does not suffer this technical limitation.

We hypothesise that bats may possess a heightened immune response when infected with virus. This allows them to quickly respond to infection and as a result is able to halt or hinder virus replication. The up-regulation of proteins involved in glycolysis indicates that there is a rapid shift towards energy production as early as 4 hpt. The down-regulation of ribosomal proteins may serve to limit the machinery available for virus replication.

## Conclusion

This is the first global proteomic analysis of the *P. alecto* cell proteome response following transfection with the viral mimic, Poly I:C. Utilising iTRAQ, we identified proteins related energy metabolism to be up-regulated while proteins associated with the ribosome to be down-regulated. The validation of some of these protein’s expression by immunodetection demonstrated that there was an induction of proteins related to energy production. Subsequent work will involve examining the proteomic response of bat cells using live viruses.

## Methods

### Maintenance of cell lines

Immortalised *Pteropus alecto* kidney (PaKiT03), lung (PaLuT02) cells [22] were maintained in D8437 Dulbecco’s Modified Eagle’s Medium Nutrient Mixture F-12 Ham (DMEM) with 15 mM HEPES, NaHCO_3_, pyridoxine and L-glutamine (Sigma-Aldrich). Human embryonic kidney (HEK293T) and human cervical cancer (HeLa) cells were maintained in Minimal Essential Medium (MEM) with 10 mM HEPES and 2 mM L-glutamine (Life Technologies). Both media used were supplemented with 10 % fetal bovine serum (FBS) (In Vitro Technologies) at 37 °C.

### Cell viability of PaKiT03 cells with Poly I:C

Cell viability was assessed following transfection with varying concentrations of Poly I:C, T24 cm^2^ flasks (Corning) were seeded with 3.5 × 10^6^ PaKiT03 cells and left at 37 °C to incubate overnight. The media were removed the following day and cell monolayers were washed with sterile phosphate buffered saline (PBS). Poly I:C (InvivoGen) solutions at 10 μg/ml, 1 μg/ml, 0.5 μg/ml and mock (control) were prepared with serum free medium according to manufacturer’s protocol. Poly I:C solutions were added to an equal volume of Lipofectamine 2000 (Invitrogen) and added to the cells and left to transfect for 6 h. Following transfection, supernatant was removed and cells were washed with PBS and fresh growth media added (DMEM supplemented with 2 % FBS). At 3, 6, 22 and 46 hpt cells were trypsinised and the cell viability was assessed with Trypan Blue staining using a hemocytometer.

### Poly I:C transfection and sampling of cells for quantitative proteomic analyses and immunodetection

PaKiT03 cells were seeded into 18 × T75 cm^2^ flasks and were incubated at 37 °C overnight. For immunodetection all cell types were seeded into individual 6 well plates. Cells were stimulated by transfection with 1 μg/ml of Poly I:C using 1 μl/ml Lipofectamine 2000 and incubated for 6 h at 37 °C. The cells were washed with PBS and placed in fresh medium.

#### Sampling for DIGE and iTRAQ

At the sampling time points 4, 8, 20 hpt the medium was removed and after a wash with sterile PBS the cells were removed with a cell scraper. Cells from one flask was resuspended in 5 ml of sterile PBS, 1 ml was removed for RNA extraction and the remaining 4 ml centrifuged and pellet resuspended in 5 ml of DIGE lysis buffer (7 M urea, 2 M thiourea, 4 % CHAPS, 30 mM Tris, pH 8.5).

#### Sampling for immunodetection

At the sampling time points 4, 8, 20 hpt the medium was removed to a 10 ml tube and centrifuged for 5 min at 1,500 × *g*. The supernatant was removed and the cells were lysed in 100 μl of 5 % SDS. Cells in the 6 well plates were lysed with 400 μl of 5 % SDS. This was then combined with the centrifuged cells in 100 μl into a fresh microfuge tube. This was boiled for 7 min at 100 °C to fragment nucleic acid.

### Real-time PCR of ISG54 following Poly I:C stimulation

The 1 ml aliquot of cells was centrifuged and resuspended in 350 μl of Buffer RLT (Qiagen). Samples were homogenised with a QIAshredder (Qiagen) and purified RNA was extracted using the Qiagen RNeasy Mini Kit (Qiagen) according to manufacturer’s instructions with on column Qiagen DNaseI digestion (Qiagen). Extracted purified RNA was resuspended in Nuclease-Free water (Promega) and concentration was determined using a NanoDrop spectrophotometer (Thermo Scientific).

The purified RNA was converted into First-Strand cDNA using SuperScript II Reverse Transcriptase (Invitrogen) according to manufacturer’s instructions. The cDNA was then used for Real-Time PCR using the SYBR Green Real-Time PCR Master Mix (Life Technologies). Primer design was completed using Primer3 with the following conditions: primer size – 20 bp, primer melting point - 60 °C, primer G-C content – 55 % and product size – 100 bp minimum, 150 bp optimum, 200 bp maximum. The cycle conditions used were holding stage step 1–95 °C for 5 min, cycling stage step 1–95 °C for 10 s, step 2–60 °C for 20 s, step 3–72 °C for 20 s for a total of 40 cycles and a default melt curve stage.

### Protein extraction and quantification for quantitative proteomics

Proteins were extracted using the 2-D Clean-Up Kit (GE Healthcare) according to manufacturer’s protocol and the purified protein pellet was solubilised in DIGE lysis buffer. Samples were quantified using the EZQ Protein Quantitation Kit (Life Technologies) with the fluorescence read at 473 nm on a Typhoon FLA 9000 (GE Healthcare). The samples were then divided equally to undergo sample preparation for each proteomic technique.

### DIGE

#### Labelling of proteins with CyDye fluorophores

50 μg of each protein sample was labelled using the minimal CyDye Fluors Labelling Kit (GE Healthcare). CyDyes were solubilised with dimethylformamide (DMF) to a concentration of 400 pmol/μl and 1 μl of CyDye was added to 50 μg of each protein sample. A dye swap was utilised with Cy3 and Cy5 between groups. An internal standard was prepared by adding equal amounts of protein and labelling with Cy2. This resulted in nine individual samples.

#### Separation of proteins by 2DE

Labelled samples in DIGE lysis buffer were reduced with 10 mg/mL dithiothreitol (DTT) then applied via cup loading to 24 cm pH 3-10NL IPG strips. Iso-electric focusing (IEF) was performed on an IPGPhor3 instrument (GE Healthcare) with the following run conditions: 1) Step and Hold – 150 V for 3 h, 2) Step and Hold – 300 V for 3 h, 3) Gradient – 1000 V 6 h, 4) Gradient – 10,000 V 1 h, 5) Step and Hold 10,000 V 5 h. After focusing for a total of 50,000 Vh, strips were incubated in equilibration buffer (6 M urea, 2 % SDS, 50 mM Tris–HCl pH 8.8, 0.02 % bromophenol blue, 30 % glycerol) with 1 % DTT for 15 min, and then another 15 min in equilibration buffer containing 2.5 % iodoacetamide. Strips were then sealed with 0.5 % agarose on top of 12.5 % SDS-PAGE gels lab cast in low fluorescence glass plates, and run using the Ettan Dalt II system (GE Healthcare) under the following conditions: Step 1) 1 W/gel for 1 h, Step 2) 17 W/gel for 5 h at 25 °C.

#### Analysis of separated protein spots with DeCyder

Following 2DE separation gels were scanned on a Typhoon FLA 9000 (GE Healthcare) using the Cy2 (488 nm), Cy3 (532 nm) and Cy5 (633 nm) excitation wavelengths. Scanned gels (triplicates for each time point) were analysed using the DeCyder software package (GE Healthcare v7.0). A spot exclusion filter was applied to spots with a spot volume of <30,000 to remove artefacts from the analysis (Fig. 2b). The remaining spots were then matched to the gel with the highest number of spots detected, designated the master gel. Only those spots that were successfully matched to the master gel were used for further analysis. Gels were matched using the internal standard with the Biological Variation Analysis (BVA) mode. Student’s *t*-test was used for statistical analysis. Protein spots were assessed as being differentially expressed if they had a p-value of < 0.05.

#### Protein spot identification

Protein spots were manually excised from gels stained with colloidal Coomassie blue. Spots were subjected to in gel trypsin digestion as described [46] for LC-MS/MS. The resulting MS/MS spectra were searched against the *P. alecto* genome [15] using MASCOT version 2.06 [47] with the following parameters: Enzyme: trypsin; Fixed modifications: Carbamidomethyl (C); Variable modifications: Oxidation (M); MS peptide tolerance: 10 ppm; MS/MS tolerance: 0.1 Da; Number of missed cleavages: up to 1.

### iTRAQ

#### Labelling of peptides

Extracted proteins from a cell lysate (100 μg) were reduced with tris-(2-carboxyethyl) phosphine (5 mM TCEP) for 1 h at 37 °C shaking at 1,150 rpm and alkylated with methyl methanthiosulfonate (MMTS 10 mM). Samples were diluted with triethyl ammonium bicarbonate buffer (TEAB 100 mM) to reduce the urea concentration to less than 1 M and proteins were digested with 2 μg of trypsin (Promega). The final volume of this solution was 133.5 μl and was incubated at 37 °C overnight with shaking at 850 rpm.

The trypsin digestion was stopped by addition of 10 μl of formic acid (Sigma). The samples were then desalted prior to labelling with a Sep-Pak C18 Plus Short Cartridge (Waters, USA). The eluted samples were evaporated in a speed vac at ambient temperature to 10 μl and TEAB was added to each tube to give a final volume of 30 μl. The pH of each sample was checked with pH 7–10 strips to ensure pH was at least pH 7.5 before addition of the iTRAQ tags. The iTRAQ tags were resuspended in isopropanol and the labelling was performed as recommended by the manufacturer. The iTRAQ labels used were 114, 115, 116, 117, 118 and 119, and the labels were cycled between replicates.

Following labelling, samples were combined into a new tube and evaporated to approximately 200 μl to remove isopropanol. Samples were then desalted on a Sep-Pak C18 Plus Short Cartridge (Waters) and vacuum concentrated to the final volume of 100 μl. Samples were then fractionated by strong cation exchange chromatography and fractions analysed by LC/MS/MS as described in [48].

#### iTRAQ analysis

Mass spectra obtained were searched against the *P. alecto* genome using ProteinPilot version 4.5with the following parameters: Sample type: iTRAQ 8plex (Peptide labelled); Cys Alkylation: MMTS; Digestion: Trypsin; Search Effort: Thorough ID. Peptide summary report was exported from ProteinPilot and filtered by confidence level according to the local 5 % false discovery rate reported by ProteinPilot. Peak lists generated by ProteinPilot were exported and used to search against the same databases using MASCOT version 2.06. The MASCOT parameters were: Enzyme: Trypsin; Fixed modifications: iTRAQ4plex (N-term), iTRAQ4plex (K), Methylthiol (C); MS peptide tolerance: 10 ppm, MS/MS tolerance: 0.1 Da, Number of missed cleavages: Up to 1. Peptide list from MASCOT was generated with a false discovery rate <1 %, determined using a concatenated reverse sequence decoy database. Proteins found using both search algorithms with a minimum of 2 peptides and identified in all three replicates were selected for further analysis as described in [48]. Proteins were assessed as being differentially expressed if the fold-change between Poly I:C/Control was ≥ 1.5.

### SDS-PAGE and immunodetection

Protein samples were analysed by immunodetection in duplicates with commercially available antibodies. Antibodies specific to β-Tublin (#2128), Eno1 (#3810) were purchased from Cell Signalling while antibodies specific to Pgam1 (NBVP1-49532) and Tpi1 (NBP1-31470) where from Novus Biologicals. Protein samples in DIGE lysis buffer were quantified using the EZQ Protein Quantitation Kit (Life Technologies) and separated under reducing conditions on precast 4–12 % Bis-Tris gels in MOPS buffer (Life Technologies). Samples were electro-transferred onto polyvinylidene fluoride (PVDF) membrane in 3-(cyclohexylamino)-1-propanesulfonic acid buffer (pH 11) with 10 % v/v methanol. The membrane was blocked over night at 4 °C with 5 % skim milk in Tris Buffered Saline with 5 % Tween-20 (TBST). The membrane was probed with appropriate species IgG conjugated with HRP. Visualisation of reactive protein bands was achieved using enhanced chemiluminescence (ECLplus, Thermo Scientific) and fluorescent detection at 473 nm on a Typhoon FLA 9000. Images of blots were imported to ImageJ (version 1.49v) and densitometry analysis was undertaken.

## Additional file

Additional file 1:
**Full list of identified proteins by LC-MS/MS and their ratio at each replicate time points determined by iTRAQ (Sheet1).** A list of the differentially regulated proteins with a mean fold-change of ≥ 1.5 (Sheet2). (XLSX 486 kb)
